# Knowledge, attitudes, and practices regarding premature ventricular contractions among patients: a cross-sectional study

**DOI:** 10.3389/fpubh.2026.1879497

**Published:** 2026-07-23

**Authors:** Zhaorui Yang, Ruifei Zhou, Fang Wang, Minqing Song

**Affiliations:** Department of Cardiology, Yichun People's Hospital, Yichun, Jiangxi, China

**Keywords:** attitudes, cross-sectional study, knowledge, practices, premature ventricular contractions

## Abstract

**Background:**

This study aimed to investigate the knowledge, attitudes, and practices (KAP) regarding premature ventricular contractions (PVCs) among patients.

**Methods:**

A cross-sectional study was conducted from March to June 2025 at Yichun People’s Hospital, enrolling patients with a confirmed diagnosis of PVCs. Demographic information and KAP scores were collected and assessed using self-designed questionnaires. KAP scores were categorized as Poor (<60%), Moderate (60–79%), and Good (≥80%) of the maximum score according to the Bloom’s criteria.

**Results:**

A total of 544 valid questionnaires were analyzed, yielding a valid response rate of 74.22%. Among the respondents, 294 (54.04%) were male, and more than half of the patients (305, 56.07%) reported not receiving any current treatment for PVCs. The knowledge, attitude, and practice scores were 11.34 ± 8.23 (range: 0–23), 26.62 ± 4.1 (range: 7–35), and 28.44 ± 7.36 (range: 8–40), respectively. Positive correlations were observed between knowledge and attitude (*r* = 0.429, *p* < 0.001), knowledge and practice (*r* = 0.380, *p* < 0.001), and attitude and practice (*r* = 0.568, *p* < 0.001). Structural equation modeling revealed that knowledge was positively associated with attitude (*β* = 0.445, *p* = 0.008) and practice (*β* = 0.235, *p* = 0.010), attitude was associated with practice (*β* = 0.527, *p* = 0.018). Furthermore, knowledge was indirectly associated with practice through attitude (*β* = 0.234, *p* = 0.009).

**Conclusion:**

PVCs patients demonstrated poor knowledge, moderate attitudes, and moderate practice concerning PVCs. The identified pathways suggest that improving PVC-related knowledge may foster positive attitudes and, in turn, healthier management practices.

## Introduction

Premature ventricular contractions (PVCs) are characterized by early depolarizations of the ventricular myocardium and represent one of the most prevalent forms of cardiac arrhythmia. These contractions arise when electrical impulses are generated within the ventricles, rather than originating from the sinoatrial node, resulting in an irregular cardiac rhythm. Epidemiological studies indicate that PVCs affect approximately 1 to 4% of the general population; however, detection rates can surpass 40% in individuals subjected to ambulatory electrocardiogram (ECG) monitoring (1, 2). The clinical implications of PVCs exhibit considerable variability. While most cases are benign and asymptomatic, increasing studies indicate that frequent PVCs may result in diminished cardiac function, an increased risk of heart failure, and a heightened likelihood of sudden cardiac death (3–5). Additionally, El Kadri et al. (6) demonstrated that the ablation of frequent PVCs in patients with nonischemic cardiomyopathy could enhance left ventricular function, implying that a considerable burden of PVCs may adversely impact cardiac performance. Recent evidence has further refined the clinical understanding and management of PVCs. Current literature indicates that the prognosis of PVCs varies substantially according to symptom burden, PVC frequency, underlying structural heart disease, and left ventricular function. Although many idiopathic PVCs are benign, frequent PVCs may contribute to PVC-induced cardiomyopathy, and timely identification of high-risk patients is therefore important. In terms of management, contemporary strategies include reassurance and observation for low-risk patients, lifestyle modification and pharmacological therapy for symptomatic patients, and catheter ablation for selected patients with frequent or symptomatic PVCs, particularly when PVC-induced cardiomyopathy is suspected. These advances have improved clinical decision-making; however, their successful implementation also depends on patients’ understanding of the disease, perceived risk, willingness to follow medical recommendations, and adherence to long-term monitoring and self-management (7, 8).

Despite advancements in diagnostic and therapeutic strategies, including enhanced pharmacological interventions (9) and catheter ablation, a significant number of patients continue to lack essential information regarding PVCs. Many individuals remain uninformed about the etiology, potential risks, warning signs, and management options associated with PVCs. This issue is clinically relevant because inadequate disease-related knowledge may affect how patients interpret symptoms, decide whether to seek medical care, adhere to medication or follow-up recommendations, and engage in lifestyle modification. In particular, patients with benign or asymptomatic PVCs may experience unnecessary anxiety, whereas those with frequent PVCs or comorbid cardiovascular disease may underestimate the need for monitoring and management (10). This deficiency in knowledge may generate unnecessary anxiety among asymptomatic patients or those not requiring treatment, while in patients with comorbidities or those needing therapy, it may lead to inadequate attention and suboptimal adherence to treatment regimens, ultimately compromising disease prognosis (11). The necessity of patient education is underscored by clinical guidelines, including the 2022 ESC Guidelines for the management of patients with ventricular arrhythmias and the prevention of sudden cardiac death (11) and the 2020 Chinese expert consensus on ventricular arrhythmias (12). These guidelines support risk-based evaluation, individualized management, and appropriate patient counseling in ventricular arrhythmia care. More recent evidence from cardiovascular health literacy research also indicates that limited health literacy is associated with poorer disease-related knowledge, medication understanding, self-management, and cardiovascular outcomes, further supporting the need for targeted patient education in PVC management (10).

The Knowledge, Attitude, and Practice (KAP) framework is a widely utilized model in health research designed to assess individuals’ comprehension, beliefs, and behaviors related to specific health issues. This framework frequently draws upon behavioral theories, including the Health Belief Model (13) and the Theory of Planned Behavior (14), thereby providing critical insights for the formulation of targeted interventions aimed at improving health outcomes (15). Although the KAP framework has been extensively utilized in the context of various heart diseases (16–18), there exists a substantial research gap regarding PVCs, despite their significant prevalence. A systematic review demonstrated that educational initiatives in cardiac rehabilitation for patients with coronary artery disease improved attendance and attitudes, indicating potential benefits for the management of PVCs (19). These findings highlight the need for KAP studies on PVCs to guide effective patient education and management strategies. Therefore, this study aims to assess the knowledge, attitudes, and practices of Chinese patients with PVC.

## Methods

### Study design and participants

This cross-sectional study was conducted on patients diagnosed with PVCs at Yichun People’s Hospital between March and June 2025. The study protocol received approval from the Medical Ethics Committee of Yichun People’s Hospital (Approval No. 2025-025), and informed consent was obtained from all participants. I confirm that all methods were performed in accordance with the relevant guidelines. All procedures were performed in accordance with the ethical standards laid down in the 1964 Declaration of Helsinki and its later amendments. Inclusion Criteria: (1) a confirmed diagnosis of PVCs through standard or ambulatory ECG, following established guideline (12); (2) being 18 years of age or older; and (3) the ability to understand the study objectives and participate voluntarily. Patients were excluded if they had other severe comorbid conditions, such as advanced malignancies or significant cardiopulmonary diseases.

### Questionnaire

The questionnaire was developed through a structured process based on a comprehensive review of existing literature and relevant guidelines on PVCs and ventricular arrhythmias (12, 20). The initial item pool was designed to cover key domains related to PVC-related knowledge, attitudes toward disease management, and self-management practices. The initial questionnaire was reviewed by five experts from the same hospital, including two cardiologists, one psychiatrist, and two medical researchers, all of whom had more than 15 years of clinical or research experience. The experts evaluated the scientific relevance, clarity, sequence, and feasibility of the items and recommended simplifying overly technical content, refining treatment-related options, and adding items on depression and emotional stress, after which the questionnaire was revised accordingly.

After preliminary revision, a pilot test involving 28 participants was conducted to evaluate the clarity, feasibility, and internal consistency of the questionnaire. The pilot study exhibited strong internal consistency, evidenced by an overall Cronbach’s *α* coefficient of 0.895. The sections pertaining to knowledge, attitude, and practice yielded Cronbach’s α coefficients of 0.880, 0.719, and 0.869, respectively, indicating acceptable to good internal consistency across all dimensions.

Confirmatory factor analysis was performed to evaluate the construct validity of the KAP questionnaire. The KMO value was 0.947, and Bartlett’s test of sphericity was significant (*p* < 0.001), indicating that the data were suitable for factor analysis. The CFA model showed a satisfactory fit, with a chi-square/degrees of freedom ratio (CMIN/DF) of 3.088, root mean square error of approximation (RMSEA) of 0.062, comparative fit index (CFI) of 0.937, Tucker–Lewis index (TLI) of 0.929, and incremental fit index (IFI) of 0.937. The detailed model fit indices and factor loadings are presented in Supplementary Tables 1 and 2, respectively, and the CFA model is shown in Supplementary Figure S1.

The finalized questionnaire, developed in Chinese, consisted of four sections. The Demographic Information section collected variables such as age, gender, education, income, and other socio-demographic characteristics. The knowledge dimension consisted of 14 single-choice items. Among them, nine items were scored as follows: well understood = 2 points, heard of = 1 point, and unclear = 0 points. The remaining five items were true/false questions, with correct answers scored as 1 point and incorrect or unclear responses scored as 0 points. The total score for the knowledge dimension ranged from 0 to 23. The Attitude Dimension consisted of seven items, assessed using a 5-point Likert scale, with responses ranging from strongly agree to strongly disagree scored from 5 to 1, yielding a total score range of 7–35. A7 was reverse-scored. The Practice Dimension included 8 items, scored on a scale from never (1) to always (5), with a total score range of 8–40. Following Bloom’s taxonomy cutoff criteria, scores for each KAP dimension were classified as Poor (<60%), Moderate (60–79%), or Good (≥ 80%) based on the percentage of the maximum possible score (21, 22).

### Questionnaire distribution and quality control

Participants were recruited utilizing a convenience sampling method. This study involved 25 healthcare professionals from the Department of Cardiovascular Medicine at Yichun People’s Hospital, including physicians and nurses, who served as trained research assistants. Before questionnaire distribution, all research assistants received a standardized 1-day training session organized by the first author, Zhaorui Yang. The training materials were developed according to the study objectives and questionnaire design, and the training content included the purpose of the study, inclusion and exclusion criteria, questionnaire distribution procedures, standardized explanations for participants, data quality control requirements, and privacy protection principles. The questionnaire was predominantly administered online via the “Questionnaire Star” (Wenjuanxing) platform, which participants accessed through QR codes. Data collection occurred across various locations affiliated with Yichun People’s Hospital and its community outreach programs. These locations included outpatient clinics for cardiovascular medicine, inpatient wards, community screening events where PVCs were confirmed through electrocardiogram, and direct consultations with patients previously diagnosed with PVCs. Trained research assistants facilitated the distribution of questionnaires and provided assistance to participants. Research assistants were present to clarify questions, ensuring that participants comprehended the material without influencing their responses.

Rigorous quality control measures were implemented to mitigate information and selection bias and to uphold data integrity. Questionnaires were systematically excluded if they indicated inattentive responses, identified through a common-sense trap question (e.g., “15 × 2 = 40”) designed to detect respondents who failed to read questions carefully or who answered randomly. Additionally, questionnaires completed in less than 60 s were excluded, as this duration was considered insufficient for adequate comprehension and thoughtful responses. Data exhibiting illogical or biologically implausible values (e.g., age = 150 years) were also identified and excluded to prevent systematic errors and anomalies in subsequent statistical analyses. Furthermore, surveys demonstrating repetitive response patterns (e.g., selecting the same option for all items) were regarded as indicative of non-authentic engagement and were subsequently excluded. Questionnaires containing contradictory answers (e.g., indicating “No underlying diseases” while simultaneously endorsing specific chronic conditions) were similarly filtered out. Finally, incomplete questionnaires were excluded from the final analysis.

### Sample size

Sample size was calculated using the formula for cross-sectional studies (23): *α* = 0.05, $\;n={\left(\frac{Z_{1-\alpha /2}}{\delta }\right)}^{2}\times p\times (1-p)$ where $Z_{1-\alpha /2}$ =1.96 when *α* = 0.05, the assumed degree of variability of *p* = 0.5 maximises the required sample size, and *δ* is admissible error (which was 5% here). The theoretical sample size was 480 which includes an extra 20% to allow for subjects lost during the study.

### Statistical analysis

All data were processed and analyzed utilizing SPSS version 26.0 (IBM Corp., Armonk, NY, USA) and AMOS version 24.0 (IBM Corp., Armonk, NY, USA). Continuous variables are presented as mean ± standard deviation (mean ± SD), while categorical variables are reported as frequency and percentage (*n*, %). For comparisons between two groups, *t*-tests were performed on normally distributed data; non-normally distributed data were analyzed using Wilcoxon-Mann–Whitney tests. For the analysis of three or more groups, ANOVA was employed for normally distributed data exhibiting equal variance, while Kruskal–Wallis tests were utilized in cases where these assumptions were not satisfied. Spearman’s rank correlation was applied to assess the relationships among knowledge, attitude, and practice scores, accommodating potential non-normality. Univariable and multivariable linear regression analyses were conducted to identify factors associated with knowledge, attitude, and practice scores. Variables with *p* < 0.1 in the univariable analyses were included in the multivariable models. Multicollinearity was assessed using the variance inflation factor (VIF). Structural equation modeling (SEM) was conducted to investigate whether attitude mediates the relationship between knowledge and practice, in accordance with the KAP model. Model fit was assessed using CMIN/DF (1–3 = excellent, 3–5 = satisfactory), RMSEA (<0.08 = good), IFI (>0.8 = good), TLI (>0.8 = good), and CFI (>0.8 = good). All statistical tests were conducted as two-sided analysis, with a significance threshold established at a *p*-value of less than 0.05.

## Results

### Demographic characteristics

A total of 733 questionnaires were initially collected, of which 544 valid questionnaires were included in the final analysis, yielding an effective response rate of 74.22%. Among the 544 participants, 54.04% were male. The majority of the participants were over 60 years (28.86%), married (70.22%), never smokers (71.88%) and non-drinkers (65.81%), with 38.79% holding a Bachelor’s degree or higher and 31.07% having junior high school or less, 37.5% engaging in occasional exercise, 75.18% having PVCs for less than 6 months, 16.54% reporting anxiety, 10.85% depression, and 49.45% having no underlying medical conditions, while hypertension (26.65%) and coronary heart disease (15.81%) were the most common comorbidities. More than half of the patients (305, 56.07%) indicated that they are not presently undergoing treatment for PVCs (Table 1).

**Table 1:** Demographic characteristics and KAP scores of PVCs patients.

Variables (*N* = 544)	*N* (%)	Knowledge, mean ± SD	*p*	Attitude, mean ± SD	*p*	Practice, mean ± SD	*p*
Total scores		11.34 ± 8.23		26.62 ± 4.1		28.44 ± 7.36	
Age (years)			0.591		0.553		0.001
18–30	138 (25.37)	12.21 ± 8.97		26.56 ± 3.78		29.64 ± 7.72	
31–40	110 (20.22)	11.93 ± 8.13		27.1 ± 3.97		26.95 ± 7.42	
41–50	61 (11.21)	10.44 ± 7.25		26.41 ± 3.74		25.9 ± 6.62	
51–60	78 (14.34)	10.65 ± 7.94		26.79 ± 3.4		28.86 ± 6.23	
> 60	157 (28.86)	10.85 ± 8.13		26.34 ± 4.84		29.2 ± 7.47	
BMI (kg/m^2^)			0.131		0.108		0.911
<18.5	28 (5.15)	10.54 ± 8.03		25.89 ± 4.19		28.11 ± 6.56	
18.5–23.9	315 (57.90)	10.77 ± 8.2		26.27 ± 4.42		28.2 ± 7.67	
24–27.9	131 (24.08)	12.7 ± 8.13		27.28 ± 3.39		28.94 ± 6.61	
≥28	70 (12.87)	11.64 ± 8.53		27.24 ± 3.59		28.71 ± 7.69	
Gender			0.879		0.546		0.803
Male	294 (54.04)	11.42 ± 8.38		26.58 ± 3.97		28.41 ± 7.05	
Female	250 (45.96)	11.24 ± 8.07		26.67 ± 4.25		28.47 ± 7.73	
Education level			0.003		0.018		0.955
Junior high school or below	169 (31.07)	11.02 ± 7.74		26.31 ± 4.43		28.06 ± 7.51	
High school/technical secondary school	84 (15.44)	8.83 ± 7.81		25.93 ± 4.39		28.64 ± 7.03	
Associate’s degree	80 (14.71)	10.95 ± 8.49		26.24 ± 4.52		28.43 ± 7.86	
Bachelor’s degree or above	211 (38.79)	12.74 ± 8.46		27.29 ± 3.41		28.67 ± 7.21	
Household monthly income (RMB)			0.417		0.189		0.962
<5,000	225 (41.36)	11.8 ± 8.38		26.18 ± 4.67		28.41 ± 7.71	
5,000–10,000	195 (35.85)	10.6 ± 8.07		26.73 ± 3.65		28.36 ± 7.15	
10,000–20,000	78 (14.34)	11.21 ± 7.93		27.19 ± 3.3		28.37 ± 6.94	
>20,000	46 (8.46)	12.46 ± 8.7		27.37 ± 3.99		29.04 ± 7.44	
Marital status			0.138		0.728		0.044
Unmarried	162 (29.78)	12.36 ± 9.18		26.64 ± 3.89		29.46 ± 7.33	
Married	382 (70.22)	10.9 ± 7.77		26.61 ± 4.19		28.01 ± 7.35	
Physical health status			0.607		0.376		0.445
Good	300 (55.15)	11.6 ± 8.78		26.84 ± 3.98		28.33 ± 7.86	
Fair	227 (41.73)	10.89 ± 7.54		26.3 ± 4.32		28.42 ± 6.74	
Poor	17 (3.13)	12.71 ± 7.19		27.06 ± 2.61		30.65 ± 6.25	
Smoking			0.651		0.002		0.139
Never	391 (71.88)	11.46 ± 8.36		26.43 ± 4.15		28.39 ± 7.52	
Former but quit	78 (14.34)	10.58 ± 8.18		26.15 ± 4		29.67 ± 6.33	
Current smoker	75 (13.79)	11.51 ± 7.66		28.08 ± 3.66		27.45 ± 7.46	
Consume alcohol			0.721		0.423		0.035
Never	358 (65.81)	11.58 ± 8.63		26.62 ± 4.23		28.68 ± 7.61	
Former but quit	93 (17.1)	11.01 ± 7.14		26.23 ± 3.83		29.2 ± 6.23	
Current drinker	93 (17.1)	10.72 ± 7.7		27.02 ± 3.84		26.74 ± 7.27	
Exercise habits			<0.001		0.157		0.050
Exercise ≥ 3 times per week	125 (22.98)	13.85 ± 8.85		26.91 ± 4.22		29.48 ± 6.99	
Exercise 1–2 times per week	99 (18.2)	12.52 ± 7.33		27.32 ± 3.14		29.55 ± 6.65	
Exercise occasionally	204 (37.5)	10.35 ± 7.94		26.39 ± 4.14		27.51 ± 7.6	
No exercise	116 (21.32)	9.36 ± 8.04		26.11 ± 4.53		28.01 ± 7.72	
Family member with cardiovascular disease			0.510		0.332		0.953
Yes	191 (35.11)	11.58 ± 8.19		26.91 ± 3.88		28.7 ± 6.84	
No	353 (64.89)	11.21 ± 8.27		26.47 ± 4.21		28.3 ± 7.64	
Time since PVC diagnosis			0.275		0.058		0.403
Less than 6 months	409 (75.18)	11.36 ± 8.38		26.83 ± 4.14		28.68 ± 7.43	
6 months to 2 years	69 (12.68)	12.17 ± 7.31		26.14 ± 3.58		28.22 ± 6.73	
More than 2 years	66 (12.13)	10.32 ± 8.25		25.8 ± 4.27		27.17 ± 7.52	
Receiving treatment			0.001		0.260		<0.001
Yes	239 (43.93)	12.53 ± 8.07		26.87 ± 3.63		30.34 ± 6.45	
No	305 (56.07)	10.4 ± 8.25		26.42 ± 4.43		26.95 ± 7.7	
Anxiety			<0.001		0.036		0.001
Yes	90 (16.54)	14.63 ± 8.15		27.41 ± 3.56		30.56 ± 6.98	
No	454 (83.46)	10.69 ± 8.1		26.46 ± 4.18		28.02 ± 7.37	
Depression			0.010		0.114		0.026
Yes	59 (10.85)	14.31 ± 8.89		27.36 ± 3.74		30.19 ± 7.72	
No	485 (89.15)	10.98 ± 8.09		26.53 ± 4.13		28.23 ± 7.3	
Regular cardiac health check-up			<0.001		<0.001		<0.001
Yes	246 (45.22)	13.14 ± 8.06		27.43 ± 3.54		30.41 ± 6.51	
No	298 (54.78)	9.85 ± 8.09		25.96 ± 4.4		26.82 ± 7.64	

### Distribution of knowledge, attitude, and practice dimensions

The knowledge, attitude, and practice were as follows: 11.34 ± 8.23 (range: 0–23), 26.62 ± 4.1 (range: 7–35), and 28.44 ± 7.36 (range: 8–40), respectively (Table 1). The analysis of knowledge dimensions indicated that the three questions with the highest “Unclear” responses were: “Wide and aberrant QRS complex indicates PVCs” (K2, 58.09%), “Criteria for malignant PVCs requiring treatment” (K7, 48.9%), and “Prevalence of PVCs detected by ECG vs. Holter” (K3, 47.79%) (Table 2). In the dimension of attitudes, there was a significant propensity among participants to comply with medical guidance, as evidenced by 34.74% strongly agreeing and 50.74% agreeing with the statement regarding their willingness to adhere to their doctor’s recommendations concerning medication and regular follow-ups (A6). Conversely, regarding the assertion “I find the treatment process for PVCs too complex to accept.” (A7), 18.01% strongly agreed and 36.03% agreed, while 15.44% disagreed and 3.49% strongly disagreed (Table 3). Responses pertaining to the practice dimension revealed that 15.99% of participants reported rarely and 9.01% never taking their prescribed medications on time to manage PVCs (P2). Additionally, 16.73% rarely and 6.25% never underwent regular ECG or other tests as recommended by their physician (P1). In contrast, adherence to dietary recommendations was comparatively higher, with only 11.95% rarely and 3.68% never following their doctor’s dietary advice (P3) (Table 4).

**Table 2:** Distribution of knowledge dimension responses.

Knowledge dimension items, *n* (%)	Very familiar	Heard of	Unclear
1. Premature ventricular contractions (PVCs) refer to premature ventricular contractions caused by ectopic foci in the ventricular myocardium below the bundle of His and its branches, also known as ventricular premature beats.	52 (9.56)	279 (51.29)	213 (39.15)
3. The prevalence of PVCs detected by routine ECG screening is approximately 1%, while detection via 24 h-48 h Holter monitoring can be as high as 40–75%.	59 (10.85)	225 (41.36)	260 (47.79)
5. Occasional premature beats generally cause no discomfort. When premature beats occur frequently or consecutively, they can reduce cardiac output and perfusion to vital organs, potentially causing symptoms like palpitations, chest tightness, fatigue, dizziness, sweating, angina, or dyspnea.	86 (15.81)	249 (45.77)	209 (38.42)
7. Patients with organic heart disease and any one of the following conditions are considered to have potentially malignant or malignant PVCs requiring treatment: ① average frequency ≥5/min; ② polymorphic or multifocal PVCs (note: exclude atrial premature beats with aberrant conduction); ③ occurring in bigeminy or trigeminy; ④ runs of three or more consecutive PVCs (brief episodes of ventricular tachycardia); ⑤ acute myocardial infarction (even occasional PVCs require prompt treatment).	69 (12.68)	209 (38.42)	266 (48.9)
8. Treatment for PVCs includes addressing the underlying cause and may involve antiarrhythmic medications.	88 (16.18)	218 (40.07)	238 (43.75)
9. For patients with premature beats, the risks and benefits of long-term antiarrhythmic drug therapy should be comprehensively evaluated.	91 (16.73)	209 (38.42)	244 (44.85)
Items of true or false	True	False	Unclear
2. The waveform with a wide and aberrant QRS complex represents a premature ventricular contraction (PVC), which is distinctly different from sinus rhythm.	183 (33.64)	45 (8.27)	316 (58.09)
4. Do you believe the following factors can cause PVCs?			
4.1 Unhealthy lifestyle factors: e.g., mental stress, excessive fatigue, excessive smoking/alcohol/caffeine intake	346 (63.6)	32 (5.88)	166 (30.51)
4.2 Various structural heart diseases: e.g., coronary heart disease, cardiomyopathy, valvular heart disease	339 (62.32)	20 (3.68)	185 (34.01)
4.3 Other factors: e.g., digitalis, quinidine, tricyclic antidepressant toxicity, electrolyte imbalances (hypokalemia, hypomagnesemia)	277 (50.92)	20 (3.68)	247 (45.4)
6. Patients without significant symptoms and no other diseases generally do not require treatment and may recover spontaneously. Patients with organic heart disease require long-term treatment.	235 (43.2)	61 (11.21)	248 (45.59)

**Table 3:** Distribution of attitude dimension responses.

Attitude dimension items, *n* (%)	Strongly agree	Agree	Neutral	Disagree	Strongly disagree
1. I believe early diagnosis and treatment of PVCs can effectively prevent heart disease.	176 (32.35)	268 (49.26)	69 (12.68)	16 (2.94)	15 (2.76)
2. I feel PVCs affect my quality of life.	150 (27.57)	274 (50.37)	89 (16.36)	20 (3.68)	11 (2.02)
3. I think delayed treatment of PVCs may lead to other serious health problems.	165 (30.33)	279 (51.29)	76 (13.97)	16 (2.94)	8 (1.47)
4. I consider medication the most effective way to control PVCs.	120 (22.06)	243 (44.67)	148 (27.21)	26 (4.78)	7 (1.29)
5. I believe lifestyle modifications (e.g., exercise, diet) are helpful for alleviating PVCs.	164 (30.15)	277 (50.92)	79 (14.52)	16 (2.94)	8 (1.47)
6. I am willing to follow my doctor’s advice regarding medication adherence and regular follow-ups.	189 (34.74)	276 (50.74)	60 (11.03)	13 (2.39)	6 (1.1)
7. I find the treatment process for PVCs too complex to accept (N)	98 (18.01)	196 (36.03)	147 (27.02)	84 (15.44)	19 (3.49)

N, negative. A7 was reverse-scored.

**Table 4:** Distribution of practice dimension responses.

Practice dimension items, *n* (%)	Always	Often	Sometimes	Rarely	Never
Do you undergo regular ECG or other tests as advised by your doctor?	104 (19.12)	155 (28.49)	160 (29.41)	91 (16.73)	34 (6.25)
Do you take prescribed medications on time to control PVCs?	119 (21.88)	153 (28.13)	136 (25)	87 (15.99)	49 (9.01)
Do you follow your doctor’s dietary recommendations (e.g., low salt, avoiding stimulants)?	147 (27.02)	186 (34.19)	126 (23.16)	65 (11.95)	20 (3.68)
Do you engage in regular appropriate exercise (e.g., walking, yoga) to maintain heart health?	114 (20.96)	175 (32.17)	160 (29.41)	75 (13.79)	20 (3.68)
Do you avoid unhealthy habits (e.g., smoking, excessive alcohol)?	173 (31.8)	161 (29.6)	116 (21.32)	66 (12.13)	28 (5.15)
When PVC symptoms worsen, do you promptly consult a doctor?	130 (23.9)	181 (33.27)	146 (26.84)	62 (11.4)	25 (4.6)
Do you regularly monitor PVC-related health indicators (e.g., weight, blood pressure)?	130 (23.9)	176 (32.35)	146 (26.84)	70 (12.87)	22 (4.04)
Do you avoid emotional stress to alleviate PVC symptoms?	124 (22.79)	174 (31.99)	153 (28.13)	71 (13.05)	22 (4.04)
If needed, would you accept radiofrequency ablation or an implantable cardioverter defibrillator (ICD)?	84 (15.44)	317 (58.27)	70 (12.87)	57 (10.48)	16 (2.94)

### Spearman correlation analysis

The significant positive correlations were found between knowledge and attitude (*r* = 0.429, *p* < 0.001), knowledge and practice (*r* = 0.380, *p* < 0.001), as well as attitude and practice (*r* = 0.568, *p* < 0.001), respectively (Supplementary Table 3).

### SEM analysis

**Figure 1:**
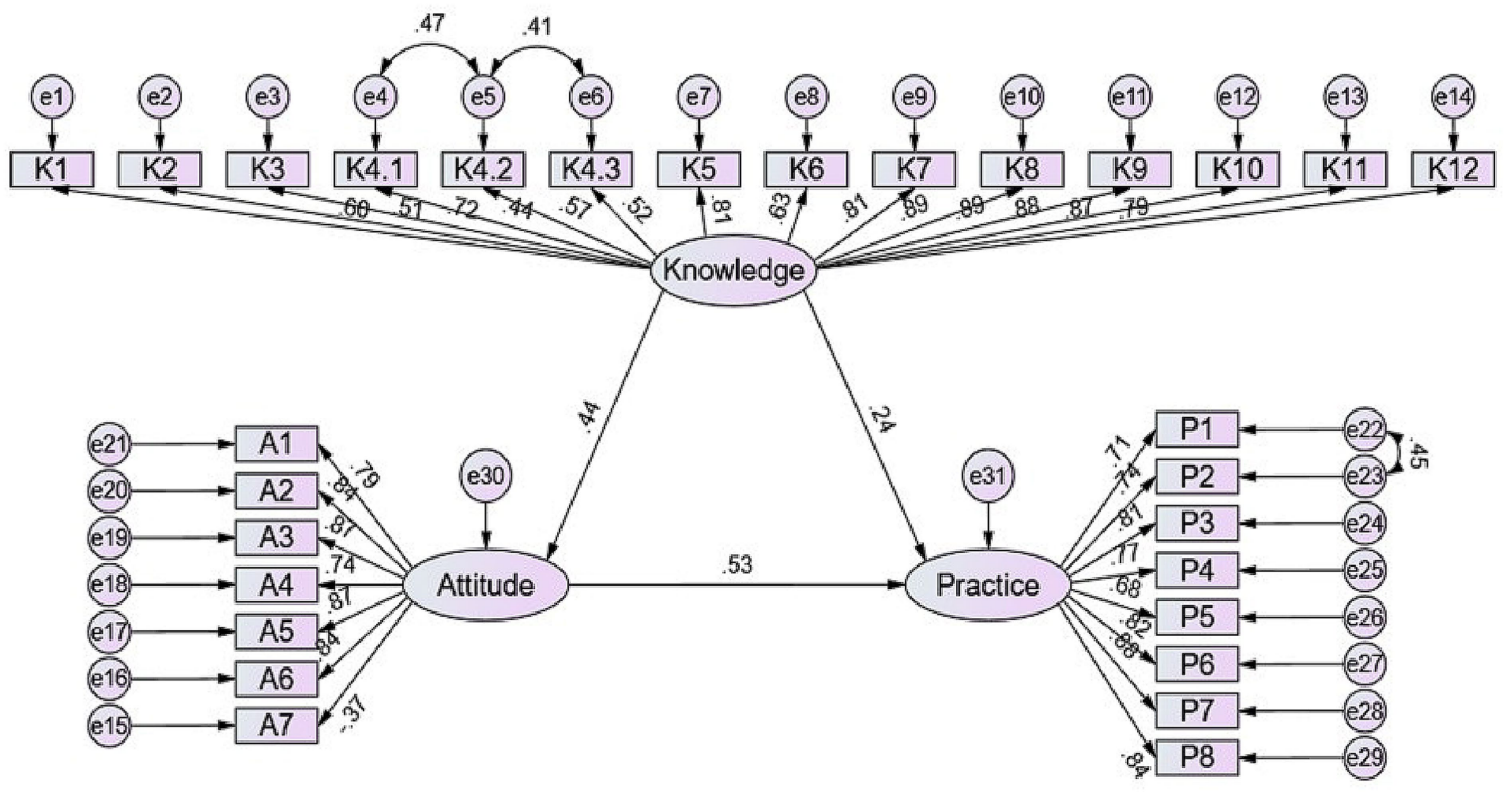
SEM model.

The SEM analysis demonstrated a satisfactory fit for the proposed model, as evidenced by a CMIN/DF value of 4.200, an RMSEA of 0.077, an IFI of 0.901, a TLI of 0.892, and a CFI of 0.901 (Supplementary Table 4). SEM analysis showed that knowledge was positively associated with attitude (*β* = 0.445, *p* = 0.008) and practice (*β* = 0.235, *p* = 0.010), attitude was positively associated with practice (*β* = 0.527, *p* = 0.018). Furthermore, knowledge was indirectly associated with practice through attitude (*β* = 0.234, *p* = 0.009) (Table 5 and Figure 1).

**Table 5:** SEM analysis and mediation effects.

Model paths	Standardized total effects		Standardized direct effects		Standardized indirect effects	
	*β* (95%CI)	*p*	*β* (95%CI)	*p*	*β* (95%CI)	*p*
Knowledge → attitude	0.445 (0.374–0.514)	0.008	0.445 (0.374–0.514)	0.008		
Knowledge → practice	0.470 (0.396–0.544)	0.012	0.235 (0.159–0.334)	0.010		
Attitude → practice	0.527 (0.408–0.615)	0.018	0.527 (0.408–0.615)	0.018		
Knowledge → practice					0.234 (0.178–0.301)	0.009

### Linear regression analysis

In the multivariable analysis, knowledge scores were independently associated with education level, exercise habits, receiving treatment, anxiety, and regular cardiac health check-ups. Attitude scores were independently associated with knowledge scores, current smoking, and regular cardiac health check-ups. Practice scores were independently associated with knowledge scores, attitude scores, age, receiving treatment, and regular cardiac health check-ups. No severe multicollinearity was observed, as all VIF values were below 3. The full results are shown in Supplementary Table 5.

## Discussion

The primary finding of this study indicates that patients with PVCs exhibit inadequate knowledge, moderate attitudes, and moderate practices concerning their condition. The application of SEM helped examine the theoretically based relationships among these dimensions, revealing significant positive associations. Notably, knowledge was associated with attitude, and attitude may play a mediating role in the association between knowledge and practice within the KAP framework.

The SEM analysis highlighted the important role of patient attitudes, which showed the strongest association with practice behavior in the proposed model. Our model demonstrated a significant association between knowledge and attitude, as well as between attitude and practice behavior. Importantly, knowledge also showed a significant indirect association with practice through attitude within the SEM framework, suggesting that patients with better cognitive understanding may be more likely to report positive attitudes and healthier practices. This finding holds clinical significance; it implies that interventions should not merely focus on disseminating information but should strategically promote positive attitudes by addressing patients’ beliefs, concerns, and perceived self-efficacy. This potential mediation pattern is consistent with established behavioral theories, such as the Health Belief Model (13) and the Theory of Planned Behavior (14), which contend that an individual’s readiness to act is related to their perceptions and beliefs, shaped by their understanding (13, 14). Consistent mediation patterns have been observed in KAP studies across various chronic conditions, emphasizing the universal significance of attitudes as a vital link between cognition and behavior (15, 16). Nevertheless, some findings did not fully follow the simple linear expectation of the KAP theory. Although patients demonstrated poor knowledge, their attitudes and practices were at moderate levels, suggesting that favorable attitudes and certain self-management behaviors may develop even when disease-specific knowledge remains insufficient. In addition, a notable discrepancy was observed within the attitude dimension: most patients expressed willingness to follow physicians’ recommendations regarding medication adherence and regular follow-ups, yet more than half also perceived the treatment process for PVCs as too complex to accept. This finding suggests that positive attitudes may coexist with perceived barriers, and that knowledge alone may not be sufficient to translate into optimal practices unless patients’ concerns, perceived treatment burden, and self-efficacy are also addressed.

A comprehensive analysis of the knowledge dimension has identified specific deficiencies, particularly in the understanding of the electrocardiographic morphology of PVCs, their prevalence across various diagnostic modalities, and the clinical criteria that classify PVCs as potentially malignant. These items were not intended to assess whether patients possessed professional-level cardiology knowledge, but rather to evaluate their basic understanding of PVC-related risk identification, the meaning of common diagnostic tests, and the circumstances under which medical attention or follow-up may be needed. This underscores a gap in patients’ understanding of clinically relevant disease information, including the significance of diagnostic findings and potential risk indicators. The prioritization of detailed medical information during routine consultations is often lacking, which may exacerbate this knowledge deficit (24). To address these identified gaps, clinical interventions should extend beyond general health advice. For instance, considering the limited understanding of ECG morphology and the criteria for categorizing PVCs as malignant, healthcare providers could develop simplified visual aids, such as infographics, brief animated videos, or interactive digital tools. These resources should elucidate fundamental ECG patterns and the factors influencing the severity of PVCs in an accessible and non-intimidating manner. Furthermore, challenges in patient education concerning other cardiac conditions, such as atrial fibrillation or heart failure, exhibit similar patterns of fragmented knowledge regarding disease mechanisms and clinical parameters. This highlights the pressing need for more customized and accessible educational interventions in the field of cardiovascular medicine (25, 26).

Despite the identified knowledge gaps, patients generally exhibited positive attitudes toward the management of PVCs, as evidenced by their strong willingness to adhere to medical recommendations. This optimistic perspective may be attributed to fundamental trust in healthcare providers, a profound desire for symptom relief, or a belief in the efficacy of medical interventions (27). However, a notable and somewhat paradoxical finding emerged: over half of the patients perceived the treatment process for PVCs as “too complex to accept.” This striking discrepancy between a high willingness to comply and a significant perception of treatment complexity constitutes a critical insight. This apparent paradox may stem from several factors. Patients may possess trust in their healthcare providers and genuinely wish to adhere to treatment, yet they may encounter practical or psychological barriers. Such barriers could include information asymmetry, wherein initial consultations fail to provide simplified explanations for long-term management; apprehension regarding medication side effects or concerns about the invasiveness of procedures such as catheter ablation; a perceived burden from ongoing lifestyle modifications (including diet, exercise, and stress management) that may feel overwhelming; or underlying anxiety associated with living with a cardiac arrhythmia, which can render any treatment more formidable (28). This finding is of paramount importance, as even with an overall positive attitude, the perception of complexity can significantly impede consistent adherence and effective self-management (29). Addressing this perceived complexity is paramount. Clinical strategies should focus on ‘simplifying the complex’ through shared decision-making, breaking down treatment plans into manageable steps, offering psychological support for anxiety, and utilizing patient navigators or peer support groups to demystify the long-term journey of PVC management.

The study observed moderate levels of engagement in actual practices. While adherence to dietary recommendations was relatively high, compliance with medication regimens and regular follow-up tests was less consistent. This variation indicates that behaviors requiring less effort or perceived immediate burden, such as dietary changes, are easier to adopt than those demanding consistent discipline, such as medication adherence or frequent medical appointments (30). This discrepancy underscores the challenge posed by perceived treatment complexity; patients may demonstrate a willingness to engage but encounter difficulties in sustaining consistent, high-effort behaviors. Furthermore, the presence of anxiety and depression among a significant portion of the patient population likely negatively affects self-management behaviors, resulting in diminished engagement with treatment protocols (31). The study primarily involved individuals who were recently diagnosed, who may experience a steep learning curve and heightened anxiety regarding their new condition, potentially influencing their initial engagement with recommended practices. Additionally, the study did not specifically assess patient participation in peer-to-peer learning or support groups, which have been shown to enhance practical knowledge and emotional resilience in other chronic conditions, and may prove beneficial in addressing perceived complexity.

This study has several limitations. First, its cross-sectional design precludes causal inference, although SEM provided useful information on potential pathways among knowledge, attitudes, and practices. Second, the single-center design and convenience sampling may limit the generalizability of the findings; therefore, future studies should consider probability-based sampling strategies or multi-center recruitment approaches. Third, the use of self-reported data may introduce social desirability bias, and participants’ subjective understanding of complex medical concepts may have influenced the accuracy of knowledge assessment. In addition, although the questionnaire demonstrated acceptable internal consistency in the pilot test and satisfactory construct validity in the CFA, the pilot test included only 28 participants and comprehensive psychometric validation was not performed. Moreover, some knowledge items involved relatively technical medical content, which may have lowered the knowledge scores and should be further refined into more patient-friendly wording in future versions of the questionnaire.

Future studies should further validate the instrument in larger and more diverse samples. Nevertheless, the formal survey included 544 valid participants, exceeding the minimum required sample size of 480. Finally, the exclusion of patients with severe comorbid conditions may further limit the applicability of the findings to patients with more complex health profiles.

These findings suggest that future interventions should address both PVC-related knowledge gaps and perceived treatment complexity. Targeted patient education, simplified risk communication, and practical self-management support may help improve attitudes and promote healthier practices. Further longitudinal and multi-center studies with more representative samples are needed to confirm these pathways and evaluate the effectiveness of educational interventions.

## Data Availability

The original contributions presented in the study are included in the article/Supplementary material, further inquiries can be directed to the corresponding author.
