# Supplementary material for: Knowledge, attitudes, and practices regarding premature ventricular contractions among patients: a cross-sectional study
Source: Front Public Health. 2026 Jul 23;14:1879497. doi: 10.3389/fpubh.2026.1879497 (PMC13442544; doi:10.3389/fpubh.2026.1879497)
Supplement: Supplementary file 1 [file Table_1.DOCX]

Questionnaire No.:

Dear Participant,

We are researchers from Yichun People's Hospital, Jiangxi Province. We sincerely invite you to participate in our research study. This study aims to understand the knowledge, attitudes, and practices (KAP) of patients with premature ventricular contractions (PVCs) regarding PVCs and their diagnosis/treatment. The findings will provide a basis for developing scientific intervention strategies, which may help improve the health of more people in the future. Your participation in this study is voluntary. This research has been reviewed and approved by the Ethics Review Board. If you agree to participate, please review the following information:

1. Please complete the questionnaire. There are no right or wrong answers; simply respond based on your actual situation. You may ask us any questions during the process. Please submit the questionnaire promptly upon completion.

2. This study involves a simple questionnaire survey. It will not cause any physical or psychological harm. However, it will involve some private information (e.g., your gender, age). We will maintain strict confidentiality, and your information will not be disclosed. Please feel assured in providing your responses.

3. As a participant, you have the right to inquire about information and research progress related to this study at any time. If you decide to withdraw from the study, please inform us. Your data will be excluded from the research results.

Finally, we sincerely thank you for taking the time to support our scientific research!

- I have read the above information and agree that the collected data may be used for scientific research.

Informed Consent Signature:

Date of Participation: Year ____ Month ____ Day ____

| **Part 1 Basic Information** | | |
| --- | --- | --- |
| 1. **Your age: ____ years.** | | |
| 2. **Your height: ____ cm.** | | |
| 3. **Your weight: ____ kg.** | | |
| **4. Your gender:** | | a. Male  b. Female |
| **5. Your education level:** | | a. Junior high school or below  b. High school / Technical secondary school  c. Associate's degree  d. Bachelor's degree  e. Master's degree or above |
| **6. Your household monthly income range:** | | a.<5000  b.5000-10000  c.10000-20000  d.>20000 |
| **7. Your marital status:** | a. Unmarried  b. Married  c. Divorced  d. Widowed | |
| **8. Your occupation:** | a. Student  b. Employed  c. Self-employed  d. Retired  e. Unemployed  f. Other | |
| **9. Your physical health status:** | a. Good  b. Fair  c. Poor | |
| **10. Do you have any of the following underlying medical conditions? (Multiple choice)** | a. Hypertension (High blood pressure)  b. Diabetes  c. Coronary heart disease (CHD)  d. Hyperlipidemia (High blood lipids)  e. Asthma / Chronic bronchitis  f. Liver disease  g. Kidney disease  h. Stroke  i. Other disease  j. No underlying medical conditions | |
| **11. Do you smoke?** | a. Never smoked  b. Former smoker (Quit)  c. Current smoker | |
| **12. Do you consume alcohol?** | a. Never drink  b. Former drinker (Quit)  c. Current drinker | |
| **13. Your exercise habits:** | a. Exercise ≥ 3 times per week  b. Exercise 1-2 times per week  c. Exercise occasionally  d. No exercise | |
| **14. Is there a family history of cardiovascular disease?** | a. Yes  b. No | |
| **15. Time since your PVC diagnosis:** | a. Less than 6 months  b. 6 months to 1 year  c. 1 year to 2 years  d. More than 2 years | |
| **16. Are you currently receiving treatment for PVCs?** | a. Yes  b. No | |
| **17. Have you ever been diagnosed with anxiety?** | a. Yes  b. No | |
| **18. Have you ever been diagnosed with depression or experienced depressive symptoms?** | a. Yes  b. No | |
| **19. Do you undergo regular cardiac health check-ups?** | a. Yes  b. No | |

| **Part 2 Understanding of Premature Ventricular Contractions (PVCs) and Their Diagnosis/Treatment Among Patients with PVCs** | | | |  |
| --- | --- | --- | --- | --- |
| **For the following statements, please indicate your level of understanding:** | | | | |
| **1. Premature ventricular contractions (PVCs) refer to premature ventricular contractions caused by ectopic foci in the ventricular myocardium below the bundle of His and its branches, also known as ventricular premature beats.** | a. very familiar | b. heard of it | c. not sure | |
| **2. The waveform with a wide and aberrant QRS complex represents a premature ventricular contraction (PVC), which is distinctly different from sinus rhythm.** | a. true | b. false | c. not sure | |
| **3. The prevalence of PVCs detected by routine ECG screening is approximately 1%, while detection via 24h-48h Holter monitoring can be as high as 40%-75%.** | a. very familiar | b. heard of it | c. not sure | |
| **4. Do you believe the following factors can cause PVCs?:** |  |  |  | |
| **4.1 Unhealthy lifestyle factors: e.g., mental stress, excessive fatigue, excessive smoking/alcohol/caffeine intake** | a. true | b. false | c. not sure | |
| **4.2 Various structural heart diseases: e.g., coronary heart disease, cardiomyopathy, valvular heart disease** | a. true | b. false | c. not sure | |
| **4.3 Other factors: e.g., digitalis, quinidine, tricyclic antidepressant toxicity, electrolyte imbalances (hypokalemia, hypomagnesemia)** | a. true | b. false | c. not sure | |
| **5.** **Occasional premature beats generally cause no discomfort. When premature beats occur frequently or consecutively, they can reduce cardiac output and perfusion to vital organs, potentially causing symptoms like palpitations, chest tightness, fatigue, dizziness, sweating, angina, or dyspnea.** | a. very familiar | b. heard of it | c. not sure | |
| **6.** **Patients without significant symptoms and no other diseases generally do not require treatment and may recover spontaneously. Patients with organic heart disease require long-term treatment.** | a. true | b. false | c. not sure | |
| **7. Patients with organic heart disease and any one of the following conditions are considered to have potentially malignant or malignant PVCs requiring treatment: ① Average frequency ≥5/minute; ② Polymorphic or multifocal PVCs (note: exclude atrial premature beats with aberrant conduction); ③ Occurring in bigeminy or trigeminy; ④ Runs of three or more consecutive PVCs (brief episodes of ventricular tachycardia); ⑤ Acute myocardial infarction (even occasional PVCs require prompt treatment).** | a. very familiar | b. heard of it | c. not sure | |
| **8. Treatment for PVCs includes addressing the underlying cause and may involve antiarrhythmic medications.** | a. very familiar | b. heard of it | c. not sure | |
| **9. For patients with premature beats, the risks and benefits of long-term antiarrhythmic drug therapy should be comprehensively evaluated.** | a. very familiar | b. heard of it | c. not sure | |
| **10. Patients with potentially life-threatening PVCs require urgent intravenous medication administration.** | a. very familiar | b. heard of it | c. not sure | |
| **11. For individuals without detected cardiac abnormalities, it needs to be repeatedly explained and reassured that PVCs do not lead to serious adverse events. If symptoms persist, appropriate antiarrhythmic medication may be considered under a doctor's guidance.** | a. very familiar | b. heard of it | c. not sure | |
| **12. Catheter ablation is recommended for PVC burden >10,000 beats/24 hours or >10% PVC burden.** | a. very familiar | b. heard of it | c. not sure | |

| **Part 3 Attitudes Toward Premature Ventricular Contractions (PVCs) and Their Diagnosis/Treatment Among Patients with PVCs** | | | | | |
| --- | --- | --- | --- | --- | --- |
| **1.** **I believe early diagnosis and treatment of PVCs can effectively prevent heart disease.** | a. strongly agree | b. agree | c. not sure | d. disagree | e. strongly disagree |
| **2.** **I feel PVCs affect my quality of life.** | a. strongly agree | b. agree | c. not sure | d. disagree | e. strongly disagree |
| **3.** **I think delayed treatment of PVCs may lead to other serious health problems.** | a. strongly agree | b. agree | c. not sure | d. disagree | e. strongly disagree |
| **4.** **I consider medication the most effective way to control PVCs.** | a. strongly agree | b. agree | c. not sure | d. disagree | e. strongly disagree |
| **5. I believe lifestyle modifications (e.g., exercise, diet) are helpful for alleviating PVCs.** | a. strongly agree | b. agree | c. not sure | d. disagree | e. strongly disagree |
| **6.** **I am willing to follow my doctor’s advice regarding medication adherence and regular follow-ups.** | a. strongly agree | b. agree | c. not sure | d. disagree | e. strongly disagree |
| **7.** **I find the treatment process for PVCs too complex to accept. (N)** | a. strongly agree | b. agree | c. not sure | d. disagree | e. strongly disagree |

| **Part 4 Behavioral Practices Regarding PVCs and Their Diagnosis/Treatment Among Patients with PVCs**  Always: Able to comply ≈100% of the time; Often: Able to comply >70% of the time; Sometimes: Able to comply 40%-70% of the time; Rarely: Able to comply 10%-40% of the time; Never: Unable to comply or able to comply <10% of the time | | | | | |
| --- | --- | --- | --- | --- | --- |
| 1. **Do you undergo regular ECG or other tests as advised by your doctor?** | a. always | b. often | c. sometimes | d. rarely | e. never |
| 2. **Do you take prescribed medications on time to control PVCs?** | a. always | b. often | c. sometimes | d. rarely | e. never |
| 3. **Do you follow your doctor’s dietary recommendations (e.g., low salt, avoiding stimulants)?** | a. always | b. often | c. sometimes | d. rarely | e. never |
| 4. **Do you engage in regular appropriate exercise (e.g., walking, yoga) to maintain heart health?** | a. always | b. often | c. sometimes | d. rarely | e. never |
| 5. **Do you avoid unhealthy habits (e.g., smoking, excessive alcohol)?** | a. always | b. often | c. sometimes | d. rarely | e. never |
| 6. **When PVC symptoms worsen, do you promptly consult a doctor?** | a. always | b. often | c. sometimes | d. rarely | e. never |
| 7. **Do you regularly monitor PVC-related health indicators (e.g., weight, blood pressure)?** | a. always | b. often | c. sometimes | d. rarely | e. never |
| 8. **Do you avoid emotional stress to alleviate PVC symptoms?** | a. always | b. often | c. sometimes | d. rarely | e. never |
| 9. **If needed, would you accept radiofrequency ablation or an implantable cardioverter defibrillator (ICD)?** | a. very acceptable | b. acceptable | c. neutral | d. unacceptable | e. very unacceptable |

**We sincerely thank you for participating in this survey. The information you provided will be invaluable for our future work!**

**Thank you for filling out our questionnaire！**

If you have any feedback or suggestions regarding this study, we would be honored to hear from you.

Comments/Suggestions: （optional）

To ensure this study achieves its goals and to facilitate future follow-ups, we would be deeply grateful if you could provide your contact information.

Your Phone Number: （optional）
