# Supplementary material for: Knowledge, attitudes, and practices regarding premature ventricular contractions among patients: a cross-sectional study
Source: Front Public Health. 2026 Jul 23;14:1879497. doi: 10.3389/fpubh.2026.1879497 (PMC13442544; doi:10.3389/fpubh.2026.1879497)
Supplement: Supplementary file 2 [file Table_2.DOCX]

**Supplementary Table 1. Model fit indices of the confirmatory factor analysis.**

| Indicators | Reference | Actual |
| --- | --- | --- |
| CMIN/DF | 1-3: Excellent, 3-5: Good | 3.088 |
| RMSEA | <0.08: Good | 0.062 |
| IFI | >0.9: Good | 0.937 |
| TLI | >0.9: Good | 0.929 |
| CFI | >0.9: Good | 0.937 |

**Supplementary Table 2. Factor loadings from the confirmatory factor analysis of the KAP questionnaire.**

|  |  |  | **Estimate** |  | **S.E.** | **C.R.** | **P** |
| --- | --- | --- | --- | --- | --- | --- | --- |
| K1 | <--- | Knowledge | 1.000 | 0.585 |  |  |  |
| K2 | <--- | Knowledge | 1.286 | 0.503 | 0.125 | 10.328 | <0.001 |
| K3 | <--- | Knowledge | 1.290 | 0.711 | 0.081 | 15.985 | <0.001 |
| K4.1 | <--- | Knowledge | 1.114 | 0.438 | 0.121 | 9.208 | <0.001 |
| K4.2 | <--- | Knowledge | 1.385 | 0.528 | 0.129 | 10.739 | <0.001 |
| K4.3 | <--- | Knowledge | 1.373 | 0.514 | 0.131 | 10.501 | <0.001 |
| K5 | <--- | Knowledge | 1.534 | 0.809 | 0.105 | 14.548 | <0.001 |
| K6 | <--- | Knowledge | 1.662 | 0.620 | 0.137 | 12.143 | <0.001 |
| K7 | <--- | Knowledge | 1.522 | 0.808 | 0.105 | 14.536 | <0.001 |
| K8 | <--- | Knowledge | 1.751 | 0.895 | 0.113 | 15.444 | <0.001 |
| K9 | <--- | Knowledge | 1.768 | 0.892 | 0.115 | 15.417 | <0.001 |
| K10 | <--- | Knowledge | 1.729 | 0.888 | 0.112 | 15.373 | <0.001 |
| K11 | <--- | Knowledge | 1.730 | 0.876 | 0.113 | 15.253 | <0.001 |
| K12 | <--- | Knowledge | 1.449 | 0.786 | 0.102 | 14.281 | <0.001 |
| A7 | <--- | Attitude | -0.513 | -0.345 | 0.065 | -7.919 | <0.001 |
| A6 | <--- | Attitude | 0.894 | 0.807 | 0.043 | 20.789 | <0.001 |
| A5 | <--- | Attitude | 0.982 | 0.842 | 0.045 | 22.051 | <0.001 |
| A4 | <--- | Attitude | 0.905 | 0.740 | 0.048 | 18.682 | <0.001 |
| A3 | <--- | Attitude | 1.028 | 0.885 | 0.044 | 23.611 | <0.001 |
| A2 | <--- | Attitude | 1.050 | 0.857 | 0.046 | 22.621 | <0.001 |
| A1 | <--- | Attitude | 1.000 | 0.793 |  |  |  |
| P1 | <--- | Practice | 1.000 | 0.711 |  |  |  |
| P2 | <--- | Practice | 1.124 | 0.743 | 0.050 | 22.472 | <0.001 |
| P3 | <--- | Practice | 1.135 | 0.843 | 0.060 | 18.791 | <0.001 |
| P4 | <--- | Practice | 1.013 | 0.769 | 0.058 | 17.361 | <0.001 |
| P5 | <--- | Practice | 0.983 | 0.681 | 0.064 | 15.393 | <0.001 |
| P6 | <--- | Practice | 1.094 | 0.811 | 0.060 | 18.305 | <0.001 |
| P7 | <--- | Practice | 1.188 | 0.881 | 0.061 | 19.493 | <0.001 |
| P8 | <--- | Practice | 1.082 | 0.806 | 0.060 | 17.995 | <0.001 |

**Supplementary Table 3. Spearman correlation analysis.**

| **Dimensions** | **Knowledge** | **Attitude** | **Practice** |
| --- | --- | --- | --- |
| **Knowledge** | 1 |  |  |
| **Attitude** | 0.429 (P<0.001) | 1 |  |
| **Practice** | 0.380 (P<0.001) | 0.568 (P<0.001) | 1 |

**Supplementary Table 4. SEM analysis fit indicators.**

| **Model fit indicator** | **Ref.** | **Measured results** |
| --- | --- | --- |
| **CMIN/DF** | 1-3 excellent，3-5 good | 4.200 |
| **RMSEA** | <0.08 good | 0.077 |
| **IFI** | >0.8 good | 0.901 |
| **TLI** | >0.8 good | 0.892 |
| **CFI** | >0.8 good | 0.901 |

**Supplementary Table 5. Univariable and multivariable linear regression analyses of factors associated with knowledge, attitude, and practice scores.**

| **Variable** | **Category** | **Univariable beta (95% CI)** | **P** | **Multivariable beta (95% CI)** | **P** | **VIF** |
| --- | --- | --- | --- | --- | --- | --- |
| Age (years) | 18-30 | Ref |  |  |  |  |
|  | 31-40 | -0.283 (-2.346, 1.780) | 0.788 |  |  |  |
|  | 41-50 | -1.768 (-4.249, 0.714) | 0.163 |  |  |  |
|  | 51-60 | -1.556 (-3.843, 0.730) | 0.183 |  |  |  |
|  | > 60 | -1.363 (-3.246, 0.520) | 0.157 |  |  |  |
| BMI (kg/m2) | <18.5 | Ref |  |  |  |  |
|  | 18.5-23.9 | 0.239 (-2.936, 3.414) | 0.883 |  |  |  |
|  | 24-27.9 | 2.167 (-1.186, 5.519) | 0.206 |  |  |  |
|  | >=28 | 1.107 (-2.493, 4.708) | 0.547 |  |  |  |
| Gender | Male | Ref |  |  |  |  |
|  | Female | -0.174 (-1.564, 1.215) | 0.806 |  |  |  |
| Education level | Junior high school or below | Ref |  | Ref |  |  |
|  | High school / Technical secondary school | -2.184 (-4.316, -0.053) | 0.045 | -2.393 (-4.437, -0.349) | 0.022 | 1.280 |
|  | Associate's degree | -0.068 (-2.235, 2.099) | 0.951 | -0.878 (-3.012, 1.256) | 0.420 | 1.340 |
|  | Bachelor's degree or above | 1.722 (0.073, 3.370) | 0.041 | 1.527 (-0.181, 3.234) | 0.080 | 1.624 |
| Household monthly income (RMB) | <5000 | Ref |  |  |  |  |
|  | 5000-10000 | -1.196 (-2.774, 0.383) | 0.138 |  |  |  |
|  | 10000-20000 | -0.590 (-2.711, 1.530) | 0.585 |  |  |  |
|  | >20000 | 0.661 (-1.950, 3.272) | 0.620 |  |  |  |
| Marital status | Unmarried | Ref |  |  |  |  |
|  | Married | -1.461 (-2.971, 0.048) | 0.058 |  |  |  |
| Physical health status | Good | Ref |  |  |  |  |
|  | Fair | -0.718 (-2.138, 0.702) | 0.322 |  |  |  |
|  | Poor | 1.103 (-2.923, 5.128) | 0.592 |  |  |  |
| Smoking | Never | Ref |  |  |  |  |
|  | Former but quit | -0.881 (-2.884, 1.123) | 0.389 |  |  |  |
|  | Current smoker | 0.049 (-1.988, 2.085) | 0.963 |  |  |  |
| Consume alcohol | Never | Ref |  |  |  |  |
|  | Former but quit | -0.573 (-2.453, 1.307) | 0.550 |  |  |  |
|  | Current drinker | -0.863 (-2.743, 1.017) | 0.368 |  |  |  |
| Exercise habits | Exercise >= 3 times per week | Ref |  | Ref |  |  |
|  | Exercise 1-2 times per week | -1.333 (-3.463, 0.797) | 0.221 | -2.417 (-4.514, -0.319) | 0.024 | 1.538 |
|  | Exercise occasionally | -3.495 (-5.293, -1.697) | <0.001 | -3.720 (-5.483, -1.957) | <0.001 | 1.710 |
|  | No exercise | -4.486 (-6.527, -2.445) | <0.001 | -4.271 (-6.286, -2.257) | <0.001 | 1.598 |
| Family member with cardiovascular disease | Yes | Ref |  |  |  |  |
|  | No | -0.374 (-1.825, 1.076) | 0.613 |  |  |  |
| Time since PVC diagnosis | Less than 6 months | Ref |  |  |  |  |
|  | 6 months to 2 years | 0.812 (-1.289, 2.913) | 0.449 |  |  |  |
|  | More than 2 years | -1.044 (-3.185, 1.098) | 0.340 |  |  |  |
| Receiving treatment | Yes | Ref |  | Ref |  |  |
|  | No | -2.128 (-3.512, -0.744) | 0.003 | -1.573 (-3.044, -0.101) | 0.037 | 1.252 |
| Anxiety | Yes | Ref |  | Ref |  |  |
|  | No | -3.948 (-5.782, -2.114) | <0.001 | -2.822 (-5.093, -0.552) | 0.015 | 1.670 |
| Depression | Yes | Ref |  | Ref |  |  |
|  | No | -3.328 (-5.537, -1.118) | 0.003 | 0.766 (-1.940, 3.473) | 0.579 | 1.663 |
| Regular cardiac health check-up | Yes | Ref |  | Ref |  |  |
|  | No | -3.293 (-4.657, -1.930) | <0.001 | -2.422 (-3.828, -1.016) | <0.001 | 1.149 |

**1.2 Attitude Dimension**

| **Variable** | **Category** | **Univariable beta (95% CI)** | **P** | **Multivariable beta (95% CI)** | **P** | **VIF** |
| --- | --- | --- | --- | --- | --- | --- |
| Knowledge |  | 0.228 (0.191, 0.266) | <0.001 | 0.214 (0.176, 0.252) | <0.001 | 1.090 |
| Age (years) | 18-30 | Ref |  |  |  |  |
|  | 31-40 | 0.542 (-0.486, 1.570) | 0.302 |  |  |  |
|  | 41-50 | -0.148 (-1.384, 1.088) | 0.814 |  |  |  |
|  | 51-60 | 0.237 (-0.902, 1.376) | 0.684 |  |  |  |
|  | > 60 | -0.220 (-1.158, 0.718) | 0.645 |  |  |  |
| BMI (kg/m2) | <18.5 | Ref |  |  |  |  |
|  | 18.5-23.9 | 0.380 (-1.196, 1.956) | 0.636 |  |  |  |
|  | 24-27.9 | 1.390 (-0.274, 3.053) | 0.102 |  |  |  |
|  | >=28 | 1.350 (-0.437, 3.137) | 0.139 |  |  |  |
| Gender | Male | Ref |  |  |  |  |
|  | Female | 0.094 (-0.598, 0.785) | 0.790 |  |  |  |
| Education level | Junior high school or below | Ref |  | Ref |  |  |
|  | High school / Technical secondary school | -0.379 (-1.444, 0.686) | 0.486 | 0.085 (-0.858, 1.028) | 0.860 | 1.280 |
|  | Associate's degree | -0.070 (-1.153, 1.013) | 0.899 | -0.225 (-1.188, 0.738) | 0.648 | 1.283 |
|  | Bachelor's degree or above | 0.986 (0.163, 1.810) | 0.019 | 0.599 (-0.143, 1.341) | 0.114 | 1.441 |
| Household monthly income (RMB) | <5000 | Ref |  |  |  |  |
|  | 5000-10000 | 0.550 (-0.233, 1.334) | 0.169 |  |  |  |
|  | 10000-20000 | 1.015 (-0.038, 2.067) | 0.059 |  |  |  |
|  | >20000 | 1.192 (-0.104, 2.488) | 0.072 |  |  |  |
| Marital status | Unmarried | Ref |  |  |  |  |
|  | Married | -0.029 (-0.783, 0.724) | 0.939 |  |  |  |
| Physical health status | Good | Ref |  |  |  |  |
|  | Fair | -0.540 (-1.246, 0.166) | 0.134 |  |  |  |
|  | Poor | 0.219 (-1.782, 2.220) | 0.830 |  |  |  |
| Smoking | Never | Ref |  | Ref |  |  |
|  | Former but quit | -0.281 (-1.268, 0.706) | 0.577 | -0.040 (-0.928, 0.849) | 0.930 | 1.069 |
|  | Current smoker | 1.645 (0.642, 2.649) | 0.001 | 1.811 (0.918, 2.703) | <0.001 | 1.044 |
| Consume alcohol | Never | Ref |  |  |  |  |
|  | Former but quit | -0.394 (-1.329, 0.540) | 0.409 |  |  |  |
|  | Current drinker | 0.401 (-0.533, 1.336) | 0.400 |  |  |  |
| Exercise habits | Exercise >= 3 times per week | Ref |  |  |  |  |
|  | Exercise 1-2 times per week | 0.411 (-0.666, 1.488) | 0.455 |  |  |  |
|  | Exercise occasionally | -0.520 (-1.429, 0.390) | 0.263 |  |  |  |
|  | No exercise | -0.800 (-1.832, 0.232) | 0.129 |  |  |  |
| Family member with cardiovascular disease | Yes | Ref |  |  |  |  |
|  | No | -0.438 (-1.159, 0.283) | 0.234 |  |  |  |
| Time since PVC diagnosis | Less than 6 months | Ref |  |  |  |  |
|  | 6 months to 2 years | -0.689 (-1.731, 0.354) | 0.196 |  |  |  |
|  | More than 2 years | -1.031 (-2.093, 0.032) | 0.058 |  |  |  |
| Receiving treatment | Yes | Ref |  |  |  |  |
|  | No | -0.452 (-1.145, 0.242) | 0.202 |  |  |  |
| Anxiety | Yes | Ref |  | Ref |  |  |
|  | No | -0.946 (-1.870, -0.022) | 0.045 | 0.143 (-0.698, 0.983) | 0.740 | 1.076 |
| Depression | Yes | Ref |  |  |  |  |
|  | No | -0.824 (-1.930, 0.282) | 0.145 |  |  |  |
| Regular cardiac health check-up | Yes | Ref |  | Ref |  |  |
|  | No | -1.470 (-2.152, -0.789) | <0.001 | -0.843 (-1.476, -0.210) | 0.009 | 1.093 |

**1.3 Practice Dimension**

| **Variable** | **Category** | **Univariable beta (95% CI)** | **P** | **Multivariable beta (95% CI)** | **P** | **VIF** |
| --- | --- | --- | --- | --- | --- | --- |
| Knowledge |  | 0.376 (0.308, 0.445) | <0.001 | 0.133 (0.066, 0.200) | <0.001 | 1.383 |
| Attitude |  | 1.027 (0.902, 1.151) | <0.001 | 0.869 (0.739, 1.000) | <0.001 | 1.294 |
| Age (years) | 18-30 | Ref |  | Ref |  |  |
|  | 31-40 | -2.690 (-4.512, -0.869) | 0.004 | -3.044 (-4.759, -1.330) | <0.001 | 2.147 |
|  | 41-50 | -3.743 (-5.935, -1.552) | <0.001 | -2.830 (-4.902, -0.758) | 0.008 | 1.935 |
|  | 51-60 | -0.786 (-2.805, 1.233) | 0.446 | -1.043 (-3.027, 0.941) | 0.303 | 2.190 |
|  | > 60 | -0.441 (-2.104, 1.222) | 0.603 | -0.447 (-2.176, 1.281) | 0.612 | 2.778 |
| BMI (kg/m2) | <18.5 | Ref |  |  |  |  |
|  | 18.5-23.9 | 0.096 (-2.755, 2.947) | 0.947 |  |  |  |
|  | 24-27.9 | 0.832 (-2.178, 3.842) | 0.588 |  |  |  |
|  | >=28 | 0.607 (-2.626, 3.840) | 0.713 |  |  |  |
| Gender | Male | Ref |  |  |  |  |
|  | Female | 0.057 (-1.186, 1.300) | 0.928 |  |  |  |
| Education level | Junior high school or below | Ref |  |  |  |  |
|  | High school / Technical secondary school | 0.584 (-1.347, 2.514) | 0.554 |  |  |  |
|  | Associate's degree | 0.366 (-1.597, 2.329) | 0.715 |  |  |  |
|  | Bachelor's degree or above | 0.614 (-0.879, 2.107) | 0.421 |  |  |  |
| Household monthly income (RMB) | <5000 | Ref |  |  |  |  |
|  | 5000-10000 | -0.054 (-1.470, 1.361) | 0.940 |  |  |  |
|  | 10000-20000 | -0.042 (-1.943, 1.859) | 0.966 |  |  |  |
|  | >20000 | 0.630 (-1.711, 2.971) | 0.598 |  |  |  |
| Marital status | Unmarried | Ref |  | Ref |  |  |
|  | Married | -1.446 (-2.795, -0.097) | 0.036 | -0.581 (-2.046, 0.885) | 0.438 | 2.034 |
| Physical health status | Good | Ref |  |  |  |  |
|  | Fair | 0.085 (-1.185, 1.355) | 0.895 |  |  |  |
|  | Poor | 2.314 (-1.286, 5.913) | 0.208 |  |  |  |
| Smoking | Never | Ref |  |  |  |  |
|  | Former but quit | 1.280 (-0.507, 3.068) | 0.161 |  |  |  |
|  | Current smoker | -0.933 (-2.750, 0.884) | 0.315 |  |  |  |
| Consume alcohol | Never | Ref |  | Ref |  |  |
|  | Former but quit | 0.520 (-1.153, 2.193) | 0.543 | 0.527 (-0.787, 1.841) | 0.432 | 1.108 |
|  | Current drinker | -1.942 (-3.615, -0.269) | 0.023 | -1.273 (-2.600, 0.054) | 0.061 | 1.130 |
| Exercise habits | Exercise >= 3 times per week | Ref |  | Ref |  |  |
|  | Exercise 1-2 times per week | 0.065 (-1.867, 1.998) | 0.947 | 0.334 (-1.198, 1.866) | 0.669 | 1.583 |
|  | Exercise occasionally | -1.965 (-3.596, -0.334) | 0.019 | -0.172 (-1.476, 1.133) | 0.797 | 1.807 |
|  | No exercise | -1.471 (-3.323, 0.380) | 0.120 | -0.046 (-1.497, 1.405) | 0.950 | 1.599 |
| Family member with cardiovascular disease | Yes | Ref |  |  |  |  |
|  | No | -0.401 (-1.698, 0.896) | 0.545 |  |  |  |
| Time since PVC diagnosis | Less than 6 months | Ref |  |  |  |  |
|  | 6 months to 2 years | -0.467 (-2.345, 1.410) | 0.626 |  |  |  |
|  | More than 2 years | -1.518 (-3.431, 0.396) | 0.121 |  |  |  |
| Receiving treatment | Yes | Ref |  | Ref |  |  |
|  | No | -3.392 (-4.607, -2.178) | <0.001 | -1.828 (-2.906, -0.751) | <0.001 | 1.295 |
| Anxiety | Yes | Ref |  | Ref |  |  |
|  | No | -2.534 (-4.187, -0.880) | 0.003 | -0.404 (-1.757, 0.950) | 0.559 | 1.146 |
| Depression | Yes | Ref |  |  |  |  |
|  | No | -1.958 (-3.942, 0.027) | 0.054 |  |  |  |
| Regular cardiac health check-up | Yes | Ref |  | Ref |  |  |
|  | No | -3.588 (-4.795, -2.381) | <0.001 | -1.636 (-2.680, -0.592) | 0.002 | 1.222 |
